# Models predicting the growth response to growth hormone treatment in short children independent of GH status, birth size and gestational age

**DOI:** 10.1186/1472-6947-7-40

**Published:** 2007-12-12

**Authors:** Jovanna Dahlgren, Berit Kriström, Aimon Niklasson, Andreas FM Nierop, Sten Rosberg, Kerstin Albertsson-Wikland

**Affiliations:** 1 Göteborg Pediatric Growth Research Center, Institute of Clinical Science, The Sahlgrenska Academy at Göteborg University, Göteborg, Sweden

## Abstract

**Background:**

Mathematical models can be used to predict individual growth responses to growth hormone (GH) therapy. The aim of this study was to construct and validate high-precision models to predict the growth response to GH treatment of short children, independent of their GH status, birth size and gestational age. As the GH doses are included, these models can be used to individualize treatment.

**Methods:**

Growth data from 415 short prepubertal children were used to construct models for predicting the growth response during the first years of GH therapy. The performance of the models was validated with data from a separate cohort of 112 children using the same inclusion criteria.

**Results:**

Using only auxological data, the model had a standard error of the residuals (SD_res_), of 0.23 SDS. The model was improved when endocrine data (GH_max _profile, IGF-I and leptin) collected before starting GH treatment were included. Inclusion of these data resulted in a decrease of the SD_res _to 0.15 SDS (corresponding to 1.1 cm in a 3-year-old child and 1.6 cm in a 7-year old). Validation of these models with a separate cohort, showed similar SD_res _for both types of models. Preterm children were not included in the Model group, but predictions for this group were within the expected range.

**Conclusion:**

These prediction models can with high accuracy be used to identify short children who will benefit from GH treatment. They are clinically useful as they are constructed using data from short children with a broad range of GH secretory status, birth size and gestational age.

## Background

Prediction models are used to determine the outcome of therapies in individual patients. Validated models for predicting individual growth responses to growth hormone (GH) treatment have been constructed for short children born appropriate for gestational age (AGA) who have a broad range of GH secretory status [1], for children with GH deficiency (GHD) [2,3], for children born small-for-gestational age (SGA) without severe GHD [4], and for girls with Turner syndrome [5]. Most models have been developed in children grouped according to birth size [4] or GH secretory status [2,3]. However, as size at birth is a continuum, and any statistically cut-off point chosen will be artificial, the development of prediction models for growth in children should be independent of birth size, especially as a broad range of the variables used improves the model [6]. The usefulness of prediction models is defined by how well they perform in practice [1,6]. It is crucial, therefore, that the model will be validated using data from a separate group of patients. The observed growth response to GH of a given child can be used as an indirect marker of GH responsiveness in this same child [7,8].

Catch-up growth during the first years of GH treatment correlates strongly with the rest of prepubertal growth [4,9] and with the total gain in height until adult height [9]. Little attention has been given to the magnitude of the catch-up growth that can be achieved when treatment is given with a GH dose optimized according to individual responsiveness. The goal of GH treatment during the first years would, therefore, be to give a dose high enough to reach the target height SD score (SDS) within a few years without producing adverse side-effects.

The aim of this study was to develop models for individual prediction of the growth response to GH during the first years of treatment, i.e., the catch-up phase, in slowly growing and/or short prepubertal children who have different GH status, gestational age and size at birth. This would facilitate the use of an evidence-based method to identify those children who will benefit from GH treatment and to individualize their GH treatment. The methodology used was non-linear multivariate regression analysis, and the decision of which variables to use was based on previous publications [1,8]. Factors known to be related closely to growth response are pretreatment growth data, auxology at the start of GH treatment, maximum peak of the spontaneous GH secretion (GH_max_)[10], and levels of insulin-like growth factor-I (IGF-I) [11] and leptin [12].

## Methods

### Study design

Data from a model group, those children beginning biosynthetic GH therapy during the period from 1986 to 1997, were used to construct the prediction models. Data from a validation group of children with the same inclusion criteria who started GH therapy during the period from 1998 to 2001, after recruiting for the model group was closed, were used to validate the prediction models.

### Patients

#### Model group

The 415 short children were born either AGA (n = 271, 59 girls) or SGA (n = 144, 34 girls). SGA is defined as a birth weight and or a birth length below -2 SDS, according to Swedish reference values [13]. Isolated idiopathic GHD was absent (n = 135) or present (n = 280), based on a cut-off level for GH_max_of 32 mU/L (10 μg/L) from either a 24-hour GH profile (24 h profile) or an arginine-insulin tolerance test (AITT).

### Validation group

The validation group consisted of a separate independent cohort of 112 children (33 girls, 79 boys) with (n = 92) or without (n = 20) isolated idiopathic GHD, of whom 34 were born SGA.

### Preterm children

A group of 36 preterm (30–36 weeks of gestation) children (Preterm model group), of whom 22 were born SGA, was studied to develop a simple general fine-tuning formula for all models. Computations were done on the model using the most readily available variables having the largest number of children included. Another preterm group of 11 children (Preterm validation group), of whom 5 were born SGA, was used for validating preterm predictions. Here, gestational age ranged from 27 to 36 weeks. Of the 47 preterm children, 24 were diagnosed as having isolated idiopathic GHD.

### Inclusion and exclusion criteria

All children were of Caucasian origin. They had an uncomplicated neonatal period, without signs of severe asphyxia (defined as an Apgar score <3 after 5 minutes) and without sepsis in the neonatal period. Height and weight have been measured since birth atneonatal units, child healthcare units and schools in Sweden. The children were well nourished and showed no clinical evidence of psychosocial disorders. Criteria for exclusion consisted of maternal history of alcohol or drug addiction, chromosomal disorders, malformations, dysmorphic features with the exception of children with Silver-Russell syndrome (n = 10), chondrodysplasia, diseases other than isolated GHD and well treated hypothyroidism. Thyroid, kidney, and liver function tests were normal. Children who missed GH injections for more than 14 days per year for the first 2 years of GH treatment were excluded. All children were prepubertal during the study period, as defined by breast stage 1 [13] or a testicular volume below 4 mL [14].

Birth weight SDS and birth length SDS used in the algorithms were calculated based on the Swedish newborn infants born 1990–94 (approximately half a million infants) from which a "healthy"" subpopulation was extracted in accordance with a former reference [15]. The improvements were that this new data set includes (a) more of very preterm infants, which stabilizes the growth curve at the lower end compared with the earlier reference; (b) gestational age evaluation was based on early ultrasound; (c) mothers who delivered by cesarean section were excluded, in order to minimize the known surplus of severely growth retarded infants rescued by intervention in the very preterm period. As a result, coefficient of variation was found to be approximately constant over the whole period. SDS were calculated using the coefficient of variation at term age (a proportionality factor equal to SD/mean) when the calculations could be based on the largest number of infants. The resulting algorithm was found to be close to estimated fetal weight [16] and was considered to describe undisturbed intrauterine growth.

As all children were prepubertal, heights were transformed into SDS for age and sex using a mathematical childhood component of the new Swedish reference [17] analogous to the ICP model of Karlberg et al [18], in order to adjust for delayed puberty. Weight SDS [17] and weight for height SDS [19] were calculated based on two published references. Gender-adjusted target height SDS was computed based on maternal and paternal height [20]. The paternal heights were measured at the clinic with few exceptions. The difference in height SDS of the child compared with its target height SDS is expressed as 'Diff SDS'.

### Pretreatment investigations

Clinical characteristics of the patients in the Model group and the Validation group are given in Table 1. The characteristics include a broad range of GH secretion and of birth size.

**Table 1:** Characteristics of all children in the Model and Validation groups. The data presented for the Auxological model (a & b) and the Endocrine models (c & d).

	**a. Model group**					**b. Validation group**				
Variables	Mean	SD	Min	Max	N	Mean	SD	Min	Max	N

**At birth**										
Gestational age (weeks)	39.6	1.3	37	42	415	39.5	1.5	37	42	112
Height SDS	-1.33	1.29	-5.8	2.0	415	-1.31	1.12	-5.4	1.5	112
Weight SDS	-1.08	1.27	-4.5	3.2	415	-1.07	1.1	-4.4	2.3	112
**Pretreatment**										
Δheight SDS during pretreat yr	0.01	0.18	-0.5	0.6	377	0.03	0.22	-0.66	0.68	101
GHmax of GH-profile	42.5	28.8	3.9	235.4	188	34.0	21.3	7.6	98.1	60
GHmax during AITT	28.6	24.4	1.7	229.4	387	21.9	14.5	1.4	76.1	95
**At GH start**										
SEX (min = girls, max = boys)			93	322	415			33	79	112
Age (yrs)	8.72	2.41	3.1	13.9	415	7.88	2.19	2.8	12.2	112
Height SDS	-2.87	0.6	-5.0	-1.2	415	-2.77	0.53	-4.2	-1.6	112
Weight SDS	-2.56	0.94	-6.2	1.8	415	-2.38	1.09	-4.9	2.6	112
Father height SDS	-1.03	1.07	-4.9	2.1	415	-0.74	1.03	-3.0	1.7	112
Mother height SDS	-1.31	1	-3.8	1.6	415	-0.99	0.96	-3.1	1.5	112
Diff SDS	-2.17	0.74	-5.0	-0.3	415	-2.29	0.72	-5.1	0.2	112
IGF SDS	-1.11	1.55	-7.4	3.0	223	-1.23	1.5	-5.3	2.7	83
Leptin (ng/mL)	3.48	2.37	1.0	27.8	216	3.97	3.75	1.7	24.1	82
**During treatment**										
GH dose (IU/kg/day)	0.11	0.03	0.07	0.25	415	0.11	0.02	0.08	0.2	112
Change in height SDS 1st yr	0.75	0.3	0.1	2.3	415	0.73	0.25	0.1	1.6	112
Change in height SDS two yrs	1.18	0.44	0.2	3.0	300	1.17	0.34	0.4	2.1	73
	**c. Model group**					**d. Validation group**				
**At birth**										
Gestational age (weeks)	39.4	1.3	37	42	140	39.6	1.4	37	42	51
Height SDS	-1.77	1.35	-5.8	1.9	140	-1.33	1.12	-4.1	1.5	51
Weight SDS	-1.39	1.36	-4.5	2.4	140	-1.19	1.02	-3.2	1.0	51
**Pretreatment**										
Δheight SDS during pretreat yr	0.07	0.17	-0.5	0.6	135	0.04	0.18	-0.6	0.4	48
GHmax of GH-profile	44.3	28.1	9.3	235.4	140	30.6	17.3	6.4	76.1	40
GHmax during AITT	37.6	22.4	3.0	124.9	113	36.7	21.9	7.6	98.1	51
**At GH start**										
SEX (min = girls, max = boys)			32	108	140			18	33	51
Age (yrs)	8.66	2.34	3.3	12.9	140	7.88	2.05	3.4	11.9	51
Height SDS	-2.86	0.61	-4.8	-1.7	140	-2.80	0.56	-4.2	-2.0	51
Weight SDS	-2.61	1.02	-5.3	1.6	140	-2.46	1.11	-4.9	1.3	51
Father height SDS	-1.04	1.08	-4.9	1.9	140	-0.71	0.91	-2.2	1.7	51
Mother height SDS	-1.39	1.04	-3.8	1.2	140	-0.88	0.87	-2.6	1.0	51
Diff SDS	-2.12	0.74	-4.6	-0.4	140	-2.37	0.77	-5.1	-0.8	51
IGF SDS	-0.82	1.32	-5.1	3.0	140	-1.49	1.54	-5.3	1.4	51
Leptin (ng/mL)	3.51	2.71	1.1	27.8	140	3.70	3.30	1.7	21.8	51
**During treatment**										
GH dose (IU/kg/day)	0.12	0.04	0.07	0.25	140	0.12	0.02	0.08	0.20	51
Change in height SDS 1st yr	0.68	0.21	0.2	1.4	140	0.77	0.23	0.4	1.6	51
Change in height SDS two yrs	1.06	0.32	0.2	1.9	104	1.21	0.33	0.7	2.1	34

**Figure 1:**
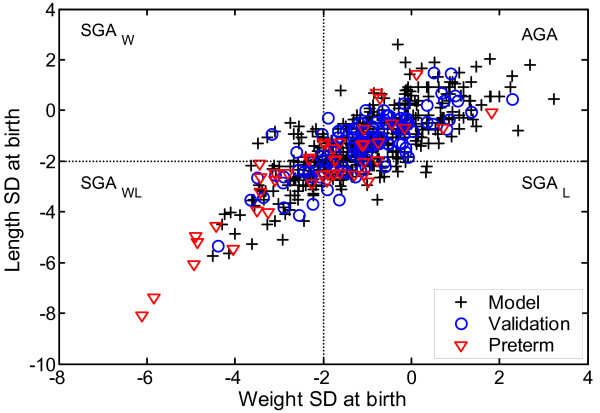
**Birth size (weight SDS versus length SDS) of the study population**. Children in the model group are shown as open circles, children in the validation group as open triangles and children born preterm as diamonds. Note the wide range of birth size and the continuous distribution.

Auxological variables in the models were (a) birth weight, (b) weight and height (SDS) at start of treatment, (c) at least one height and weight measurement between birth and the start of treatment, (d) maternal height SDS and (e) paternal height SDS. The characteristics of the SGA and preterm children are shown in Table 2. Figure 1 shows the wide distribution in birth size of all the studied children.

**Table 2:** Characteristics of children included born either SGA (a and b) or preterm (c and d). The data is presented for the Auxological model.

	**a. Model group**					**b. Validation group**				
Variables	Mean	SD	Min	Max	N	Mean	SD	Min	Max	N

**At birth**										
Gestational age (weeks)	39.4	1.3	37	42	144	39.2	1.5	37	42	34
Height SDS	-2.58	0.93	-5.8	-0.5	144	-2.55	0.84	-5.4	-0.9	34
Weight SDS	-2.25	0.89	-4.5	0.4	144	-2.19	0.80	-4.4	-0.6	34
**Pretreatment**										
Δheight SDS during pretreat yr	0.02	0.19	-0.5	0.6	134	0.03	0.19	-0.3	0.7	32
GHmax of GH-profile	47.8	27.8	11.7	146.9	77	38.5	23.6	14.2	98.1	18
GHmax during AITT	34.6	26.2	3.0	160	133	24.3	17.8	5.9	76.1	28
**At GH start**										
SEX (min = girls, max = boys)			34	110	144			14	20	34
Age (yrs)	8.1	2.51	3.1	13.3	144	7.8	2.20	3.2	11.4	34
Height SDS	-3.04	0.65	-4.8	-1.5	144	-2.94	0.54	-4.2	-2.0	34
Weight SDS	-2.79	1.05	-6.2	1.6	144	-2.71	0.98	-4.9	0.0	34
Father height SDS	-1.07	1.17	-4.9	1.9	144	-0.93	1.00	-2.8	1.7	34
Mother height SDS	-1.39	1.09	-3.6	1.2	144	-1.28	0.97	-3.1	1.0	34
Diff SDS	-2.29	0.82	-4.6	-0.4	144	-2.27	0.83	-5.1	-0.8	34
IGF SDS	-0.81	1.46	-7.4	3.0	87	-1.02	1.64	-4.0	2.7	25
Leptin (ng/mL)	3.65	3.24	1.2	27.8	92	3.12	1.05	1.7	5.3	25
**During treatment**										
GH dose (IU/kg/day)	0.11	0.03	0.07	0.22	144	0.11	0.02	0.09	0.18	34
Change in height SDS 1st yr	0.7	0.28	0.1	2.0	144	0.64	0.19	0.1	1.0	34
Change in height SDS two yrs	1.1	0.41	0.2	3.0	108	1.03	0.32	0.4	1.9	26
	**c. Model group**					**d. Validation group**				
**At birth**										
Gestational age (weeks)	34.4	1.7	30	36	36	34.4	2.5	27	36	11
Height SDS	-2.57	1.96	-8.1	0.7	36	-1.75	1.55	-4.0	1.4	11
Weight SDS	-2.18	1.66	-6.1	0.7	36	-1.78	1.6	-3.5	1.8	11
**Pretreatment**										
Δheight SDS during pretreat yr	-0.05	0.22	-0.4	0.5	32	0.03	0.1	-0.1	0.2	11
GHmax of GH-profile	44.1	25.9	14.3	134	22	28.6	10.5	15.4	48.9	10
GHmax during AITT	28.6	15.1	8.5	70.4	33	38.3	17.4	8.8	63.8	10
**At GH start**										
SEX (min = girls, max = boys)			7	29	36			5	6	11
Age (yrs)	7.89	2.91	2.8	14.6	36	6.69	1.32	4.8	9.0	11
Height SDS	-2.84	0.59	-4.1	-1.5	36	-2.61	0.36	-3.2	-2.0	11
Weight SDS	-3.01	0.89	-5.2	-0.7	36	-2.40	1.14	-5.2	-0.2	11
Father height SDS	-1.16	0.88	-3.4	0.4	36	-0.40	1.21	-2.8	1.3	11
Mother height SDS	-1.02	0.89	-3.2	1.2	36	-0.68	1.26	-3.4	1.6	11
Diff SDS	-2.2	0.72	-3.5	-1.0	36	-2.36	0.52	-3.3	-1.3	11
IGF SDS	-1.54	1.19	-5.4	0.2	26	-1.12	1.41	-3.3	0.7	11
Leptin (ng/mL)	3.25	1.16	2.1	7.1	21	3.96	1.73	2.5	8	9
**During treatment**										
GH dose (IU/kg/day)	0.11	0.03	0.08	0.19	36	0.14	0.03	0.10	0.18	11
Change in height SDS 1st yr	0.68	0.25	0.4	1.2	36	0.71	0.16	0.5	1.0	11
Change in height SDS two yrs	1.1	0.39	0.6	2.2	28	1.18	0.16	0.9	1.4	8

The endocrine investigations were performed during the pre-treatment year, and included a GHstimulation test, Arginin Insulin Tolerance Test, (AITT), as described previously [21]. Also, a spontaneous 24 h GH profile with integrated samples taken every 20 minutes [22] was obtained from 188 children (40 girls, 148 boys) in the Model group and 60 children (20 girls, 40 boys) in the Validation group. Three other children in the Validation group had a 12 h night-time GH profile [10]. At the start of treatment, blood samples for leptin were taken in the morning, and for IGF-I in the afternoon, from 200 children in the Model group and from 48 in the Validation group. The clinical characteristics of the children from whom only auxological information was used, were not different from the children in whom serum IGF-I and leptin or the maximum level of GH_24 h _24 h GH profiles and maximum level of GH_AITT _GH_AITT _were obtained (see Table 1).

### Treatment

All children were treated with a daily dose of GH ranging between 25 and 66 μg/kg based on the body weight (kg) of the child. The exact dose was adjusted every 3 months.

### Biochemical measurements

All analyses were performed at the lab of Växthuset, accredited no 1899.

*GH *concentrations were measured using a time-resolved immunofluorometic assay (Wallac, Finland), with the WHO First International Reference Preparation (IRP) 80/505 as the standard [23]. If another method or an earlier standard was used, the GH concentrations were transformed to comparable levels using transformation factors derived in our laboratory [24]. Accredited no:1899.

*IGF-I *was measured by an IGFBP-blocked radioimmunoassay (RIA) without extraction and in the presence of an approximately 250-fold excess of IGF-II (Mediagnost, Tübingen, Germany) [25]. IGF-I values are expressed as SDS [26]. Accredited no:1899.

*Leptin *was measured by RIA (Linco Research Inc, St Charles, MO, USA). The detection range of the assay was 0.22 to 100 ng/ml and, the intra-assay coefficients of variation were 7.0% at 2.4 ng/mL and 4.9% at 14.0 ng/ml. The corresponding values for the interassay coefficients of variation were 9.6% and 6.7% [27]. Accredited no:1899.

### Statistics

#### General considerations about model fitting in prediction models

We present analyses of the standard error of the residuals SD_res_, because they are independent of the numerical value of the predicted growth response. The R^2 ^analysis is too sensitive for the range of the predicted extremes, (i.e., very high or very low responders)[6]. The model is clinically valid if the SD_res _for the validation group of patients is in the same range as the group of patients used to derive the model.

#### Modelling

The technique used is described as non-linear data fitting (empirical curve fitting) and empirical testing. The non-linear approach was chosen because a non-linear relationship was found between the growth response and other variables. Overfitting was prevented, by selecting stepwise subsets of non-linear transformed original variables that gave the best overall prediction result. As the growth response curve is non-linear, a non-linear correction for differences in measurement time (1 year ± 3 months) was developed. The modeller had no access to the data from the validation group and testing of the final prediction models with the validation group, was performed by another statistician. A computer program for calculation of the prediction was constructed for each of the five models presented in the results section, using the algorithms presented in Additional file 1. This program was used for prediction of the growth response for the children in the validation group and compared with the observed growth response for each child.

### New derived variables

In the set of possible predictor variables we have added some derived variables, based on auxological measurements, which were not included in our previous model [1]. A well-known derived variable for the individual characterization of growth is, for example, the height SDS. For a child with normal growth, height SDS remains more or less constant over time. In children with disturbed spontaneous growth, there will be a systematic increase or decrease in SDS. By extending the height SDS with an extra individual regression weight it was possible to describe normal as well as disturbed spontaneous growth [8]. This extra regression weight is computed by fitting the observed pretreatment growth in SDS with a non-linear 'disturbed growth SDS' function. The intercept of this function gives an indication of the level of normal growth and the slope gives a measure of the amplitude and direction of disturbed growth. In the earlier model (ref 1 in the manuscript) the theoretical extrapolation of the 'untreated' growth curve in height SDS beyond the start of GH treatment was computed with an exponential curve fitted with two height measurement points: the first at one year before start of treatment and the second at start of treatment. This exponential curve gave an estimate of the growth curve if the child was not treated, but the form of the curve was a best guess and not based on data fitting over several years. The "untreated end level SDS" can be compared with the target height SDS or other auxological data and result in relevant prediction variables for predicting the response to GH.

### GH dose in the models

In most biological systems, the response to dose is logarithmic, as it is for GH [28]. However, a linear dose relationship has been reported [4,29] in the GH-dose range normally used (17–100 μg/kg/day). We therefore constructed models with either linear or logarithmic dose transformations. As the SD_res _were similar, we present only the linear dose models.

### Ethics

The GH treatment studies were approved by the Ethical Committees of the Medical Faculties of the Universities of Göteborg, Lund, Linköping, Uppsala and Umeå and of the Karolinska Institute. Informed consent was obtained from all children (if old enough) and their parents.

## Results

### A. Prediction models

The variables available for modelling and those selected are indicated in Table 3. Either length SDS or weight SDS at birth could be used in the models with the same result in terms of SD_res_. As measurement of birth length is not performed in some countries, the weight SDS was used in the models. Five prediction models were developed. The auxological model was constructed first. The different endocrine variables were then added to produce the endocrine models. Results are calculated for the first 1, 2 and 3 years of GH treatment (Table 4). Results for the SGA and preterm children are shown in Table 5. The daily dose of GH/kg body weight was constant in time and included in all the models with variable name DoseG. The equations for the models are given in Additional file 1.

**Table 3:** Variables used in the different models.

	***Auxological model***	***Endocrine models***			
		***IGF+leptin***	***GH_AITT_+IGF+leptin***	***GH_24 h_***	***GH_24 h_+IGF+leptin***

***At birth***					
Gestational age (weeks)	x	x	x	x	x
Length					
Weight	x	x	x	x	x
Gender	x	x	x	x	x
					
***Pretreatment growth***					
Height & weight					
After 9 months	x	x	x	x	x
					
***At GH start***					
Age at start	x	x	x	x	x
Height	x	x	x	x	x
Weight	x	x	x	x	x
					
**Parental heights**					
Mother's height	x	x	x	x	x
Father's height	x	x	x	x	x
					
***Biochemical***					
GH_max _24 h				x	x
GH_max_AITT			x		
IGF-I		x	x		x
leptin		x	x		x
**GH dose**	x	x	x	x	x

**Table 4:** SD_res _results in the total Model group (left) and total Validation group (right) giving the first, second and third year response. SDstandRes gives the ratio SDres/SDresModelgroup, 2*cm 3 y gives the 2 SD prediction interval in cm at 3 years of age and 2*cm 7 y gives this interval at 7 years of age.

	***Model group***					***Validation group***	
**Models**	n	SD_res_	SDstand_Res_	2*cm 3 y	2*cm 7 y	n	SD_res_

**1 yr response**							
A. *Auxological model*	415	0.231	1	1.7	2.4	112	0.230
B. *Auxological+endocrine models*							
IGF+leptin	199	0.191	1	1.4	2.0	82	0.209
GH_AITT_+IGF+leptin	172	0.191	1	1.4	2.0	71	0.205
GH_24 h_	188	0.164	1	1.2	1.7	60	0.165
GH_24 h_+IGF+leptin	140	0.154	1	1.1	1.6	51	0.156

**2 yrs response**							
A. *Auxological model*	305	0.340	1	2.5	3.5	77	0.351
B. *Auxological+endocrine models*							
IGF+leptin	154	0.266	1	1.9	2.7	55	0.290
GH_AITT_+IGF+leptin	138	0.260	1	1.9	2.7	45	0.280
GH_24 h_	133	0.276	1	2.0	2.8	43	0.289
GH_24 h_+IGF+leptin	105	0.246	1	1.8	2.5	34	0.261

**3 yrs response**							
A. *Auxological model*	191	0.432	1	3.2	4.5	27	0.470
B. *Auxological+endocrine models*							
IGF+leptin	109	0.327	1	2.4	3.4	17	0.370
GH_AITT_+IGF+leptin	98	0.329	1	2.4	3.4	12	0.358
GH_24 h_	86	0.353	1	2.6	3.6	14	0.400
GH_24 h_+IGF+leptin	73	0.305	1	2.2	3.1	9	0.343

**Table 5:** SD_res _results for children born SGA, giving the first, second and third year response. Model group (left) and total Validation group (right). SDstandRes gives the ratio SD_res_/SD_res_Modelgroup, 2*cm 3 y gives the 2 SD prediction interval in cm at 3 years of age and 2*cm 7 y gives this interval at 7 years of age.

	***Model group***					***Validation group***	
**Models**	n	SD_res_	SDstand _Res_	2*cm 3 y	2*cm 7 y	n	SD_res_

**1 yr response**							
A. *Auxological model*	144	0.221	0.96	1.6	2.3	34	0.211
B. *Auxological+endocrine models*							
IGF+leptin	83	0.188	0.98	1.4	1.9	25	0.177
GH_AITT_+IGF+leptin	72	0.186	0.97	1.4	1.9	20	0.185
GH_24 h_	77	0.169	1.03	1.2	1.7	18	0.137
GH_24 h_+IGF+leptin	68	0.167	1.08	1.2	1.7	17	0.107

**2 yrs response**							
A. *Auxological model*	112	0.326	0.96	2.4	3.4	27	0.343
B. *Auxological+endocrine models*							
IGF+leptin	69	0.284	1.07	2.1	2.9	21	0.295
GH_AITT_+IGF+leptin	61	0.268	1.03	2.0	2.8	16	0.270
GH_24 h_	60	0.292	1.06	2.1	3.0	15	0.297
GH_24 h_+IGF+leptin	55	0.266	1.08	1.9	2.7	13	0.280

**3 yrs response**							
A. *Auxological model*	77	0.440	1.02	3.2	4.5	9	0.459
B. *Auxological+endocrine models*							
IGF+leptin	49	0.338	1.03	2.5	3.5	7	0.361
GH_AITT_+IGF+leptin	43	0.321	0.98	2.3	3.3	4	0.326
GH_24 h_	42	0.388	1.10	2.8	4	6	0.380
GH_24 h_+IGF+leptin	39	0.336	1.10	2.5	3.5	5	0.361

#### I. Auxological model

This model was created using growth data collected between the first years of life and the start of treatment, together with parental heights. The SD_res _for the first-year response to GH treatment in the Model group was 0.23 SDS.

#### II. Endocrine models

*1*. *IGF-I+leptin*: In this model, IGF-I SDS and leptin levels at the start of treatment were added to the auxological model. The accuracy of the prediction was improved (i.e. the SD_res _was reduced) giving an SD_res _for the Model group of 0.19 SDS.

*2*. *GH*_*AITT*_+*IGF-I+leptin*: The peak value of the GH_AITT _and IGF-I and leptin at the start of treatment were added to the auxological model, giving a similar SD_res _as above (0.19 SDS). Thus, addition of GH_AITT _did not improve the model.

*3*. *GH*_*24 h*_: Information from the spontaneous GH profile was obtained and the peak value (found to be most informative) was selected, and used together with the auxological model, giving an SD_res _of 0.16 SDS.

*4*. *GH*_*24 h*_+*IGF-I+leptin*: When IGF-I and leptin at the start of treatment were added to the *GH*_*24 h *_model, the SD_res _decreased to 0.15 SDS, indicating that this is the most accurate model.

### B. Validation of the models

The results from the validation indicated that the prediction accuracy was consistent with the results from all the Model groups, indicating that all the models are valid statistically (see Table 4, right column).

**Figure 2:**
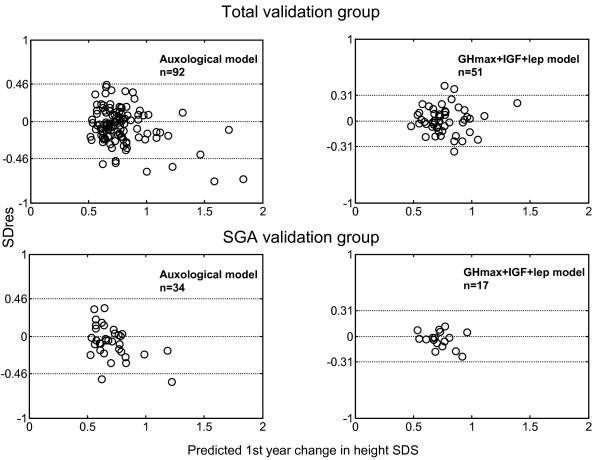
**The SD of the residuals (SD_res_) in relation to the predicted growth response in the validation group**. Individual circles show each child. The 2 SD interval for the model group is indicated by broken lines. Note that 2 SD of the validation group for both models are within the confidence interval, despite the more narrow SD_res_.

The observed growth in relation to the predicted growth response is visualized for each child in the Validation groups in Figure 2. Approximately 95% of the children have an observed individual growth response within the 2 SD_res _for both models.

#### C. Children born SGA or preterm

Prediction and validation in short children born SGA were within the expected range for all models (see Table 5). For the group born preterm, predictions were within the expected range with the least accurate auxology model. For the endocrine models, a simple fine-tuning computed on a preterm model group (n = 22) was needed to obtain results with an SD_res _in the same range. The results of fine-tuning the models are shown in Table 6.

**Table 6:** SD_res _results for children born preterm, giving the first and second year response. Model group (left) and total Validation group (right). SDstandRes gives the ratio SD_res_/SD_res_Modelgroup, 2*cm 3 y gives the 2 SD prediction interval in cm at 3 years of age and 2*cm 7 y gives this interval at 7 years of age.

	***Model group***					***Validation group***	
**Models**	N	SD_res_	SDstand_Res_	2*cm 3 y	2*cm 7 y	n	SD_res_

**1 yr response**							
A. *Auxological model*	36	0.185	0.80	1.4	1.9	11	0.147
B. *Auxological+endocrine models*							
IGF+leptin	21	0.159	0.83	1.2	1.6	9	0.124
GH_AITT_+IGF+leptin	18	0.161	0.84	1.2	1.7	8	0.117
GH_24 h_	22	0.124	0.75	0.9	1.3	10	0.128
GH_24 h_+IGF+leptin	16	0.125	0.81	0.9	1.3	8	0.120

**2 yr response**							
A. *Auxological model*	28	0.273	0.80	2.0	2.8	8	0.179
B. *Auxological+endocrine models*							
IGF+leptin	19	0.228	0.86	1.7	2.4	7	0.094
GH_AITT_+IGF+leptin	16	0.263	1.01	1.9	2.7	6	0.087
GH_24 h_	16	0.176	0.64	1.3	1.8	7	0.158
GH_24 h_+IGF+leptin	14	0.164	0.67	1.2	1.7	6	0.102

## Discussion

We here present growth prediction models to be used when GH treatment is given to short prepubertal children independent of GH status, gestational age or birth size. This study presents validated prediction models with the most accurate estimates of the growth response to GH treatment available. The model with the best accuracy using auxological and endocrine variables has a 2 SD_res _of 0.30 SDS, corresponding to a prediction interval of ± 1.2 cm in a 4-year-old boy. On the other hand, using only auxological data, it is still possible to predict the individual growth response during the first year with a narrow prediction interval of ± 1.9 cm in the same 4-year-old boy (2 SD_res _of 0.46 SDS). This is possible due to non-linear modelling and the inclusion of new mathematically derived growth variables, based on clinical knowledge. In the future, new prediction models adding data such as bone markers [3], genetics or proteomic variables may improve the accuracy of the models further, since the information for computing the best model presented here is not always available in clinical practice worldwide. Therefore we think it is useful to search for the construction of simpler models with growth data more directly related to the GH growth responsiveness. It will give more insight in the underlying processes and can give better prediction results when certain information like the parents height or 24-hour GH profile is lacking.

As the models described here were developed in a group of children with a wide range of values in the variables used, these models tend to be more robust [6] than those restricted by arbitrary cut-offs in, for example, birth size, gestational age/preterm birth or GH secretion. Most other published models have been developed from data from children selected according to birth size [1-4] or GH status [2-4]. In clinical practice this might be inconvenient, as the models will not cover a large number of the children who might be treated with GH. We also developed models based exclusively on data from children born SGA (data not shown). These were as accurate (same SD_res_) as those with a broader range in birth size.

The value of our multivariate algorithms is strengthened by the use of one group of children to develop the models, and use of a separate group of children for validation. The algorithms, therefore, fulfil the criteria of prediction models [6]. The non-linear approach used in the models may have contributed to the more narrow prediction interval compared with models constructed by others using a linear approach [2-5]. Our simplest auxological model gives a better prediction interval than previous models for GH-deficient children and for short children born AGA, who have various GH secretory capacities [1]. Also, we observe a similar prediction interval for children born SGA, as in models constructed only for children born SGA (± 2.2 cm in a 6.6-year-old child) [4].

Compared with the KIGS prediction model for children born SGA [4], we obtain a broader prediction range in SDS and cm/year. This will provide a more appropriate prediction of high responders. The risk of overfitting (i.e. constructing too accurate predictions in a model group, which are not reliable and therefore give bad validation results) has been debated [30]. In our study, overfitting was prevented, by selecting stepwise subsets of transformed original variables that gave the best overall prediction result. The prevention of overfitting is proven by the consistent validation results of our models. The low SD_res _for the different models is a sign of the low ratio of extreme residuals, i.e. high predictive accuracy for most individual children. The greater the accuracy in the model the smaller the prediction error, and the lower the risk of making a sub-optimal clinical decision about a treatment. The models presented serve as a practical clinical tool for selecting children for successful GH treatment.

In the prediction models we use indirect variables for fat (weight and leptin), liver (IGF-I) and bone (linear growth). The finding that the GH_max _value from the spontaneous profile was the most informative variable, and more predictive than the GH peak in the provocation test, was known previously [1], and reflects both the higher reproducibility of the former and its greater relevance to growth [31,32]. The GH value obtained during the spontaneous profile and growth response to GH therapy reflects the interplay between these two variables. We have reported [10] that nighttime GH_max _has nearly the same predictive value for the growth response to GH treatment as the maximum level from 24 h GH sampling. The profile GH_max _was informative to the point that adding IGF-I and leptin data to the 24 h model improved the prediction interval only slightly. These variables, however, improved the prediction interval if added to the auxological model. The effect of GH on fat tissue seems to be closely related to the growth response to GH treatment [12,33]. Leptin can be used as an indirect marker of responsiveness, in fat tissue either mirroring nutritional status or as a sign of GHD.

In clinical practice, if it is not possible to perform a spontaneous GH profile, IGF-I and leptin are informative, although not as useful as adding the GH_max _from the profile. To make a more informed selection of those children who will benefit from GH treatment, a decision can be made among the more simple auxological model, keeping in mind the sub-optimal SD_res_, or the more precise endocrine models with GH_max _or IGF-I and leptin. Although the latter models are more costly in terms of the investigations required, they are improved for selecting children for treatment.

Authorities in Europe and the USA have approved GH treatment for short children born SGA, regardless of their GH secretory status. Various treatment regimens have been studied with high-dose GH treatment, based on the observation of elevated circulating GH concentrations in SGA newborns [34,35]. A correlation has been observed between the GH dose and the early growth response [4]. Some of these children, however, have satisfactory catch-up growth with a dose of 33 μg/kg/day, especially those who start treatment at a young age [9,35,36]. These children have been observed to have significant variability in their growth response to treatment with fixed doses of GH. In this group, therefore, GH dosing should be individualized using prediction models that give an estimate of responsiveness.

## Conclusion

The models presented here are independent of birth size and provide the highest prediction accuracy available. They serve as a tool to identify those children who may benefit from GH treatment, and to help choose the optimal GH dose during the first years of treatment in order to optimize the individual catch-up growth response.

## Abbreviations

AGA appropriate for gestational age

AITT arginine-insulin tolerance test

Diff SDS the intra-family difference in SDS (i.e. the difference in height SDS of the child compared to his/her MPH SDS)

GH_AITT _the estimated maximal GH level from AITT

GHD GH deficient

GH_max _the estimated maximal GH level from the spontaneous GH profile

IGF-I insulin-like growth factor-I

IGF-II insulin-like growth factor-II

IGFBP-3 IGF binding protein 3

MPH mid-parental height SDS

SDS SD score

SD_res _root mean square error of the residuals

SGA small for gestational age

GH_24 h _maximal GH level during a 24 h profile

GH_AITT _peak GH at AITT

## Competing interests

Kerstin Albertsson Wikland (KAW) declares that she have an unrestricted research grant from Pharmacia/Pfizer. Berit Kristrom (BK) has received reimbursement for expert consultant work for Pfizer but has no shares or received any salary. Andreas FM Nierop (AFMN) works for Muvara, Multivariate Analysis of Industrial and Research Data Statistical Consultation, The Netherlands.

## Authors' contributions

JD, BK, AN, SR, AFMN and KAW have all given substantial contribution to conception and design, analysis and interpretation of the data. AN have performed all the modelling work. JD, BK, AN, AFMN and KAW have been involved in drafting the manuscript and have revised it critically for important intellectual content. JD, BK, AN, SR, AFMN and KAW have given final approval of the version to be published.

## Pre-publication history

The pre-publication history for this paper can be accessed here:



## Supplementary Material

Additional file 1 Algorithms Click here for file
