# Supplementary material for: Models predicting the growth response to growth hormone treatment in short children independent of GH status, birth size and gestational age
Source: BMC Med Inform Decis Mak. 2007 Dec 12;7:40. doi: 10.1186/1472-6947-7-40 (PMC2246105; doi:10.1186/1472-6947-7-40)
Supplement: Additional file 1 — Algorithms [file 1472-6947-7-40-S1.doc]

Additional file 1:

Algorithms for the models

**Predicted Change in Height SDscore** (**ΔHSDS) during GH Treatment**

ΔHSDS(t, RS_Predicted_) = F_BG_(t) + RS_Predicted_ *F_CU_(t) *= non-linear longitudinal description of predicted GH growth response curves,* *where t = treatment time (years)*

RS_Predicted_ = RS_Taux_, RS_Tigflep_, RS_Taittigflep_, RS_T24Haux_ or RS_T24Higflep_, *= predicted response scores defined below for five models*

F_BG_(t) = ln (1 + t*(0.205 - 1/(1 + 2.1*t))) *= baseline growth response function*

F_CU_(t) = t/(t+0.894) *= hyperbolic catch-up function*

RS = the response score is an individual factor combining the effect of dose and individual responsiveness, as will be described in more detail in Kriström et al. (manus in preparation)

**Total auxology model (Taux):**

RS_Taux_ = F(Theight18cint) + F(DhcTHEISD) + F(Dwhfit18) + F(CeIwh) + F(OGPHEISD) + F(DFMHEISD) + *= predicted response score*

F(Wsdbirth) + 3.69799107*DoseG - 0.194459196

RS_Taux_ = RS_Taux_ + F_Tpreterm_(agecgG)*(GA<37) + 0.316920199 *= finetuning the response score for Preterms*

GA<37 = Binary variable for selecting preterms with *Gestational Age* < 37 weeks

F(Variable): p1-p4 = p1*Variable + p2*(p3 + (Variable – p4)^2)^0.5

F(Variable): p1-p4 = F(Theight18cint): Ta1-Ta4, F(DhcTHEISD): Ta5-Ta8, F(Dwhfit18): Ta9-Ta12, *The estimated coefficients p1-p4 of each function F(Variable) are given after the colon*

F(CeIwh): Ta13-Ta16, F(OGPHEISD): Ta17-Ta20, F(DFMHEISD): Ta21-Ta24,

F(Wsdbirth): Ta25-Ta28, F_Tpreterm_(agecgG): Ta29-Ta32

Theight18cint = F_BGint_(18- agecgG) + RSc_Theight18_* F_CUint_(18- agecgG), = i*ntegral of response level needed to reach*

*Theight SD score at 18 years of age corrected for disturbed growth*

F_BGint_(t) = Σ_k_ (t*a_k_*log(1+t*a_k_ *(0.205-1./(1+2.1.*t*a_k_)))), for k=1,2,3, and [a_1_ a_2_ a_3_]=[2/3 1/6 1/12] *= approximation of integral of F_BG_(t)*

F_CUint_(t) = t+0.894*log(0.894/(t+0.894)) *= integral of F_CU_(t)*

ageG = age at start of GH treatment

agecgG = G(ageG) *= ageG corrected for Gestational Age*

G(age) = age+((0.5+GA) -40)/52 *= age corrected for Gestational Age*

*0.5 week is added, because Gestational Age is categorized in whole weeks and therefore the corresponding average age for children in week x is x+0.5*

RSc_Theight18_ = ((THEISD- Hcsdg) - F_BG_(18- agecgG))/ F_CU_(18- agecgG) *= RS needed to reach Theight at 18 years*

Hcsdg = (Hsdg – (CeIh-CmIh)*(0.5 – 0.5*CmI(ageG)))/(0.5 + 0.5*CmI(ageG)) *= Hsdg corrected*

Hsdg = Hsd(ageG)

Hsd(age) = (Observed height in cm - T(age))/Tsd(age)

T(age) = I(age) + C(age)

I(age) = k_i1_*(1-e^-ki2*(G(age)+ k3)^)

C(age) = k_c1_*(G(age)+ k_3_) - k_c2_*(G(age)+ k_3_)^2^

Tsd(age) = Isd(age) + Csd(age)

Isd(age) = I_cv_ x I(age)

Csd(age) = C_cv_ x C(age),

CmI(age) = (Csd(age) - Isd(age))/Tsd(age)

Omit patient: if (3*age_first measurement after 9 months_) > ageG *for all longitudinal height or weight measurements*

W(age_i_) = 0 *for all measurements i with G(age_i_) <= 9 months (0.75)*

1/n1 *for all n1 measurements i around age 1 within range 0.75 < G(age_i_) <= 1.75*

1/n2 *for all n2 measurements i around age 2 within range 1.75 < G(age_i_) <= 2.5*

1/ny *for all ny measurements i around age y (y=3,4,..) within range (y-0.5) < G(age_i_) <= (y+0.5)*

A_i_ = W(age_i_)*(CmI(age_i_)-CmI(ageG))*(Hsd(age_i_)-Hsd(ageG))

B_i_ = W(age_i_)*(CmI(age_i_)-CmI(ageG))^2^

CmIh = (Σ_i_ A_i_)/( Σ_i_ B_i_), *for all longitudinal measurements i with age_i_ <= ageG*

CeIh = Hsdg - CmIh*CmI(ageG)

THEISD = (THEIcm- th_1_))/ th_2_) *= target height as predicted by parental heights in Luo et al. (20)*

THEIcm = th_3_+ th_4_*(0.5*(MHEIcm + FHEIcm))

MHEISD = (MHEIcm- th_1_)/th_2_

FHEISD = (FHEIcm- th_1_)/th_2_

DhcTHEISD = THEISD – Hcsdg

Dwhfit18 = WHsdfit18 - WHsdg

WHsdg = WHsd(ageG): *weight for height in Karlberg&Albertsson-Wikland (19)*

Wsdg = Wsd(ageG): *weight in Albertsson-Wikland et al. (17)*

WHsdfit18 = CeIwh + CmIwh*CmI(iG(age)) *for age=18*

iG(age) = age-((0.5+GA) -40)/52 *= inverse of G( age)*

CmIwh = (Σ_i_ Awh_i_)/( Σ_i_ Bwh_i_), *as CmIh, for all longitudinal weight for height measurements i with age_i_ <= ageG*

CeIwh = WHsdg - CmIwh*CmI(ageG)

Wsdbirth = weight at birth, accepted BMC Niklasson reference based on (15) and (16)

OGPHEISD = Other gender parent height SD score

DoseG = GH dose (IU/kg.day) *is kept constant during treatment*

DFMHEISD = FHEISD - MHEISD

**Total auxology + IGF-I + leptin model (Tigflep):**

RS_Tigflep_ = 0.526365582*RS_Taux_ + 1.495049995*DoseG + F(LogLEPG) + F(IGF_SDg) - 0.467253240 *= predicted response score*

F(Variable): p1-p4 = F(LogLEPG): Ti1-Ti4, F(IGF_SDg): Ti5-Ti8

LogLEPG = log10(1+Leptine_at start of GH treatment_)

IGF_SDg = IGF_at start of GH treatment_ SD score

**Total auxology + GHmax AITT + IGF-I + leptin model (Taittigflep):**

RS_Taittigflep_ = 0.498045656*RS_Taux_ + 1.952846536*DoseG + F(LogGHMAXG) + F(LogLEPG) + *= predicted response score*

F(IGF_SDg) + 0.408978736

F(Variable): p1-p4 = F(LogGHMAXG): Tt1-Tt4, F(LogLEPG): Tt5-Tt8, F(IGF_SDg): Tt9-Tt12

F(LogGHMAXG)= log10(1+ GHmax AITT_at start of GH treatment_)

**Total auxology + 24h GH model (T24Haux):**

RS_T24Haux_ = 0.530579181*RS_Taux_ + 2.575166372*DoseG + F(LogGHMX24HG) + 1.638768091 *= predicted response score*

F(Variable): p1-p4 = F(LogGHMX24HG): Tg1-Tg4

LogGHMX24HG = log10(1+GHMX24H_at start of GH treatment_)

**Total auxology + 24h GH + IGF-I + leptin model (T24Higflep):**

RS_T24Higflep_ = 0.385381630*RS_Taux_ + 2.674325949*DoseG + F(LogGHMX24HG) + F(LogLEPG) + *= predicted response score*

F(IGF_SDg) + 0.323969689

F(Variable): p1-p4 = F(LogGHMX24HG): Tgi1-Tgi4, F(LogLEPG): Tgi5-Tgi8, F(IGF_SDg): Tgi9-Tgi12

**Response Score Index (Estimated responsiveness)**

RSi(DoseG, RS) = RS – B_DoseG_*(DoseG – 0.1)

*with Regression weight B_DoseG_ based on model T24Higflep as follows: B_DoseG_ = 0.38538163.*3.69799107+2.674325949 = 4.099463775*

*and with predicted or observed response score RS as applied below*

RSi_Pred_(DoseG, RS_Pred_) = RS_Pred_ – B_DoseG_*(DoseG – 0.1) *the best choice for RS_Pred_ is RS_T24Higflep_*

RSi_Obs_(DoseG, RS_Obs_)= RS_Obs_ – B_DoseG_*(DoseG – 0.1)

RS_Obs_ = (ΔHSDS_Obs_(t) – F_BG_(t)) / F_CU_(t) = *observed response score, where t = observed treatment time (years)*

**Coefficients:**

Ta1-Ta32=[ 0.004901991 0.006277855 193.589469183 20.704767301; 0.165924156 0.230719729 1.597412670 3.951094691;

0.211329516 0.340835258 0.175120176 0.415361877; 0.119869990 0.082867301 0.881916834 0.423032194;

0.086730476 -0.056892641 1.064252302 -2.330226463; 0.022415362 0.169731795 1.695939172 0.151165935;

0.030033051 -0.031096758 1.614769494 0.192775853; -0.180537748 0.250750104 8.453185693 5.756169371]

Ti1-Ti8=[1.177941334 -0.661786892 0.021801823 0.467961236; -0.152977029 0.134736195 2.178403201 -2.526294121]

Tt1-Tt12=[-0.378907753 -0.303147178 0.082781428 1.623498925; 0.781609650 -0.521215713 0.022352019 0.640616382;

-0.146176702 0.133667337 2.217376113 -2.539489296]

Tg1-Tg4=[-1.009128653 0.818618216 0.061398697 1.319354314]

Tgi1-Tgi12=[-0.282198638 0.364222975 0.048782465 1.817334318; 0.933385614 -0.489157440 0.023787832 0.464374692;

-0.050288573 0.049302021 1.734273429 -0.486134573]

**Coefficients for girls:**

k_i1_ = 62.54785757042, k_i2_ = 1.823677860659, k_3_ = 0. 6627671903241, k_c1_ = 9.949738642531, k_c2_ = 0.2514723125035,

th_1_ =167.59, th_2_ = 6.08, th_3_ = 37.85, th_4_ = 0.75,

I_cv_ = 0.02987709478516, C_cv_ = 0.05321530312222, cmi_1_ = 1.53622413352, cmi _2_ = 5.725824397339, cmi _3_ = 1.625768448342

**Coefficients for boys:**

k_i1_ = 65.04702921079, k_i2_ = 1.833525449549, k_3_ = 0. 6494334970962, k_c1_ = 9.405436598935, k_c2_ = 0.2125041669303,

th_1_ =180.43, th_2_ = 6.58, th_3_ = 45.99, th_4_ = 0.78,

I_cv_ = 0.03013392384305, C_cv_ = 0.05377684289457, cmi_1_ = 1.537990152142, cmi _2_ = 6.184553263652, cmi _3_ =1.611054697992

**Variable Description**

GA Gestational age in weeks

THEISD Target height SD score

MHEISD Mother height SD score

FHEISD Father height SD score

DFMHEISD Father - mother height SD score

OGPHEISD Other gender parent height SD score

agecgG Age (y, corrected for gestational age)

Hsdg Height SD score at start of GH treatment

Wsdg Weight SD score at start of GH treatment

WHsdg Weight for height SD score at start of GH treatment

Wsdbirth Weight SD score at birth

CeIh Location of disturbed growth curve for height

CmIh Slope of disturbed growth curve for height

CeIwh Location of disturbed growth curve for weight for height

CmIwh Measure of the amplitude and direction of disturbed growth curve for weight for height

WHsdfit18 Untreated endlevel in weight for height SD score at 18 years of age extrapolated from disturbed growth curve

Dwhfit18 Untreated weight for height SD endlevel at 18 years - Weight for height SD score at start

Hcsdg Height SD score corrected for disturbed growth

DhcTHEISD THEISD - Height SD score corrected for disturbed growth

RScTheight18 Response level needed to reach Theight SD score at 18 years of age corrected for disturbed growth

Theight18cint Integral of response level needed to reach Theight SD score at 18 years of age corrected for disturbed growth
